# Coexistence of charge and ferromagnetic order in fcc Fe

**DOI:** 10.1038/ncomms10949

**Published:** 2016-03-14

**Authors:** Pin-Jui Hsu, Jens Kügel, Jeannette Kemmer, Francesco Parisen Toldin, Tobias Mauerer, Matthias Vogt, Fakher Assaad, Matthias Bode

**Affiliations:** 1 Physikalisches Institut, Experimentelle Physik II, Universität Würzburg, Am Hubland, D-97074 Würzburg, Germany; 2 Institut für Theoretische Physik und Astrophysik, Universität Würzburg, 97074 Würzburg, Germany; 3 Wilhelm Conrad Röntgen-Center for Complex Material Systems (RCCM), Universität Würzburg, Am Hubland, D-97074 Würzburg, Germany

## Abstract

Phase coexistence phenomena have been intensively studied in strongly correlated materials where several ordered states simultaneously occur or compete. Material properties critically depend on external parameters and boundary conditions, where tiny changes result in qualitatively different ground states. However, up to date, phase coexistence phenomena have exclusively been reported for complex compounds composed of multiple elements. Here we show that charge- and magnetically ordered states coexist in double-layer Fe/Rh(001). Scanning tunnelling microscopy and spectroscopy measurements reveal periodic charge-order stripes below a temperature of 130 K. Close to liquid helium temperature, they are superimposed by ferromagnetic domains as observed by spin-polarized scanning tunnelling microscopy. Temperature-dependent measurements reveal a pronounced cross-talk between charge and spin order at the ferromagnetic ordering temperature about 70 K, which is successfully modelled within an effective Ginzburg–Landau ansatz including sixth-order terms. Our results show that subtle balance between structural modifications can lead to competing ordering phenomena.

## Abstract (web summary)

The coexistence of different magnetic and electronic phases often occurs in materials with complex chemical compositions, allowing for the study of competitive, collaborative, or emergent phenomena. Here, the authors demonstrate such behaviour in ultrathin Fe films on a Rh(001) substrate.

## Graphical abstract

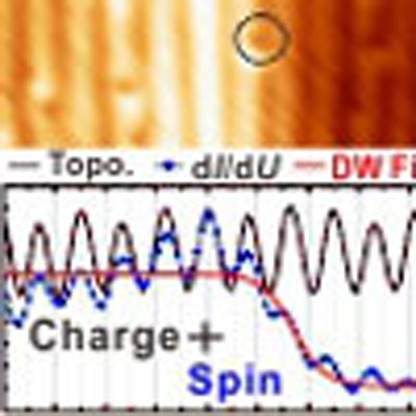

In the recent past, competing order phenomena such as the interplay between spin and charge order in copper- and iron-based superconductors^1^,^2^,^3^, the magnetic modulation-induced emergence of spontaneous polarization in multiferroics^4^,^5^,^6^,^7^ or the coexistence of magnetism and superconductivity at the interface of oxide heterostructures^8^,^9^,^10^ have intensively been investigated. In these materials, subtle changes of the chemical composition or external stimuli may eventually lead to non-trivial emergent excitations at quantum critical points between lowest energy states^11^.

Although iron (Fe) is usually considered the prototypical ferromagnetic material, it exhibits strong correlations between atomic, orbital and magnetic spin structure, which result in a rich variety of interesting magnetic properties. Bulk Fe crystallizes in a body-centre-cubic (bcc) crystal structure and shows robust ferromagnetism (FM) with a Curie temperature *T*_C_=1,043 K (ref. 12). For low-dimensional Fe ultra-thin films and nanostructures, however, various magnetic ground states and non-trivial spin textures have been theoretically predicted, including non-magnetic^13^, non-collinear antiferromagnetic (AFM) ordering^14^,^15^, incommensurate spin–density wave^16^, helical spin spiral^17^ and magnetic skyrmions^18^.

Recent advanced experimental studies^19^,^20^,^21^,^22^ indicate that these apparently contradicting reports are caused by the fact that the magnetic ground state of Fe is highly sensitive to the interplay of electronic hybridization and structural instabilities in reduced dimensions. In particular, the magnetism of ultra-thin pseudomorphic Fe films on face-centre-cubic (fcc) Rh(001), which has been subject of several investigations^23^,^24^,^25^,^26^,^27^,^28^, appears to be strongly influenced by the competition between ferromagnetic order in bcc *α*- and antiferromagnetism in fcc *γ*-Fe, as well as electronic hybridization of the film's 3*d* states with the substrate's 4*d* states^25^. Although the monolayer exhibits an AFM *c*(2 × 2) spin structure^25^,^26^,^28^, films with a local thickness of 2 and 3 atomic layers (ALs) are ferromagnetically ordered with the easy axis of magnetization along the surface normal^27^,^28^. It has been speculated that the competition between AFM and ferromagnetic order in tetragonally distorted films may lead to low-energy excitations or competing phase transitions^25^.

In this study, we report on the observation of a phase coexistence phenomenon in pseudomorphic Fe double-layer films grown on Rh(001). Our scanning tunnelling microscopy (STM) and spectroscopy (STS) measurements reveal that different order phenomena, that is, charge and ferromagnetic spin order, coexist at low temperatures far below the respective phase transitions. Interestingly, we observe a remitted reduction of the charge-order parameter *ϕ* at the ferromagnetic Curie temperature, indicating a cross-talk between charge and spin order. This behaviour has been successfully modelled by Ginzburg–Landau (GL) calculations. We speculate that this cross-talk may be mediated by electronic states at or very close to the Fermi level. As the system investigated here is structurally much more simple than other materials with coexisting order phenomena, it may become a model system and allow for a better understandings of competing order phenomena.

## Results

### Coexistence of charge and ferromagnetic spin order

**Figure 1:**
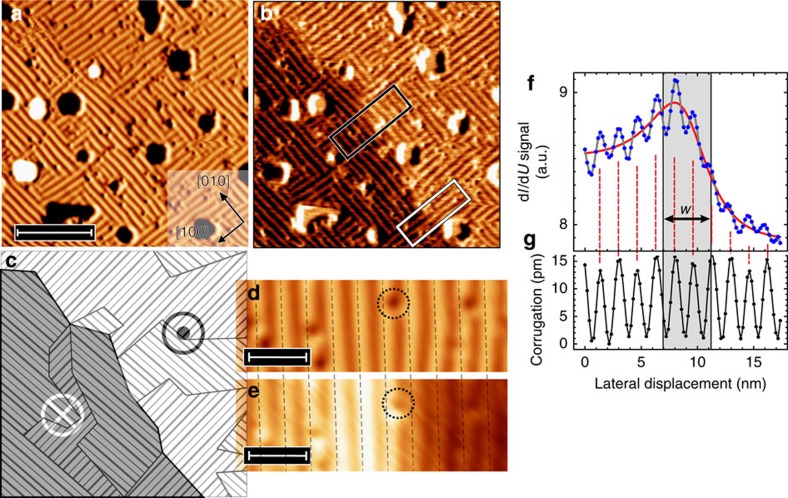
Coexistence of magnetic domains and a striped phase. (**a**) Topography and (**b**) the simultaneously measured spin-resolved d*I*/d*U* map of (1.95±0.02) AL Fe/Rh(001) showing out-of-plane magnetic domains (scan parameters: *U*=−0.7 V, *I*=500 pA, *T*=5 K). Scale bar, 15 nm. The white and black boxes mark regions where the stripe superstructure is oriented parallel (**q**_1_) or perpendicular (**q**_2_) to the magnetic domain wall, respectively. (**c**) Schematic drawing of the magnetic domain and stripe patterns observed in **b**. (**d**,**e**) Zoomed-in topographic and d*I*/d*U* image of a location similar to the one shown in the black box in **b** (*U*=−1.0 V, *I*=500 pA). Scale bars, 3 nm. (**f**,**g**) Line sections obtained from **e** and **d**, showing spin-resolved d*I*/d*U* signal together with topographic corrugation. Fitting the domain wall (red line) as defined in ref. 29 gives a wall width *w*=(4.32±0.35) nm. It is noteworthy that the presence of the domain wall does not significantly influence the modulation of the stripe superstructure, as indicated by the equally spaced dashed red lines.

Figure 1a,b shows the topography and the differential conductivity d*I*/d*U*, respectively, of a (1.95±0.02) AL Fe film on Rh(001) resolved by using spin-polarized STM (SP-STM) at a temperature *T*=5 K. An almost perfectly closed double layer is obtained, with a few holes and some tiny triple-layer islands as the only imperfections. Both data sets were measured simultaneously using a Cr-coated probe tip with out-of-plane magnetic sensitivity^29^. The magnetic contrast is particularly well visible in Fig. 1b, which shows the tunnelling magnetoresistance contrast of two domains with opposite perpendicular magnetization as signalled by the dark and bright regions in the lower left and upper right of the image.

The Curie temperature *T*_C_ of this double layer turns out to be very low. Although *T*_C_ of a 3.0-AL Fe film on Rh(001) amounts to ∼320 K, a steep linear decrease has been observed towards thinner films^24^. For the ferromagnetic double layer, no magnetic signal could be detected down to *T*=97 K (ref. 24). Linear extrapolation to an Fe coverage of 2.0 AL^24^ suggests a Curie temperature below 80 K. In fact, our temperature-dependent SP-STM measurements confirm this finding (see Supplementary Fig. 1). It has been speculated that these properties may be related to the above-mentioned FM–AFM competition, which potentially results in a very low Curie temperature and/or excited magnetic states at relatively low excitation energies^25^. As we will show below, the situation appears to be even more complex, with different competing ordering phenomena at work.

Interestingly, Fig. 1a,b also reveal that the out-of-plane FM spin order of the Fe double layer on Rh(001) coexists with a periodic one-dimensional superstructure that consists of stripes along the [100] and [010] directions of the substrate. For clarity, the coexisting magnetic domain and stripe patterns observed in Fig. 1b is schematically represented by dark/bright background and differently oriented periodic lines in Fig. 1c, respectively. The periodicity of the stripes amounts to 1.48±0.22 nm, corresponding to superstructures with wave vectors **q**_1_=(2*π*/*a*)(0.26±0.03, 0, 0) and **q**_**2**_=(2*π*/*a*)(0, 0.25±0.03, 0), where *a* is the Rh(001) atomic lattice constant of 3.80 Å.

Detailed analysis indicates that at the measurement temperature of 5 K, there is no significant correlation between the stripe pattern and the magnetic domain structure. For example, the black and white boxes in Fig. 1b mark surface areas where the stripe superstructure is oriented parallel (**q**_2_) or perpendicular (**q**_1_) to this domain wall, respectively. In neither case the presence of the domain wall seems to have any significant influence on the stripes.

This impression is also confirmed by the analysis of an area with a configuration similar to the black box in Fig. 1b, that is, with stripes along the [010] directions (**q**_2_) across a magnetic domain wall. Figure 1d,e shows the topography and the d*I*/d*U* map, respectively. An individual defect is marked by circles in both images. Figure 1f presents line sections drawn along the long axes of these images. Comparison of the peak position of the stripe superstructure in both the topographic and the spin-resolved d*I*/d*U* channel shows no indication for any changes across the domain wall (see dashed red vertical lines in Fig. 1e).

### Electronic structure of charge-ordered phase

**Figure 2:**
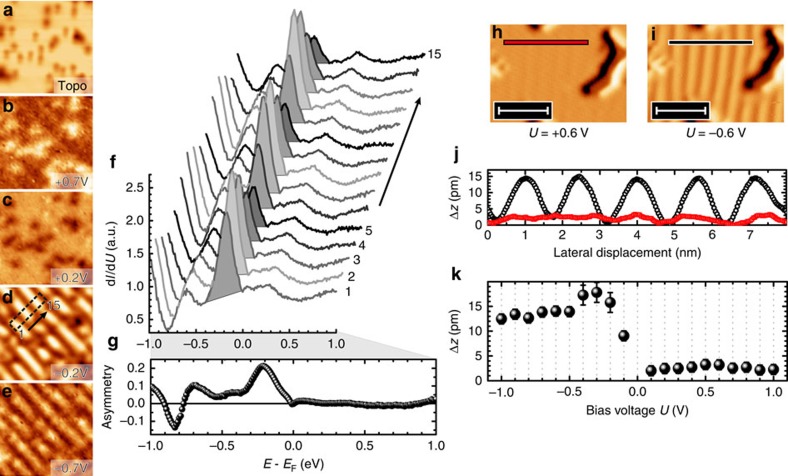
STS and bias voltage-dependent corrugation. (**a**) Topography of the Fe double layer on Rh(001) and (**b**–**e**) d*I*/d*U* maps taken at the indicated bias voltages from constant-separation STS data (setpoint parameters: *U*=+1.0 V, *I*=500 pA). The stripe pattern is only visible in the occupied energy range. Image sizes are 15 × 15 nm^2^. (**f**) Tunnelling spectra measured along the box in **d**. The peak at −0.2 V is more intensive on the bright stripes than between them. (**g**) Plot of the electronic asymmetry as a function of bias voltages. Although the electronic asymmetry is negligible in the empty states, it becomes maximal in the occupied states at about −0.2 V. (**h**,**i**) Topographic images taken at *U*=+0.6 V and −0.6 V. Scale bars, 5 nm. (**j**) Averaged line sections measured along the red and black lines. (**k**) Bias dependence of the corrugation. Error bars are given by standard deviation of corrugation peak heights measured at corresponding bias voltages.

To unravel the physical origin of the stripes, we have performed STS measurements to probe the local density-of-states of the region shown in Fig. 2a (topography). Figure 2b–e shows a series of d*I*/d*U* maps extracted from this data set at some representative bias voltages *U*. Obviously, the appearance of stripes strongly depends on *U*. Although the stripes cannot be detected within the signal-to-noise ratio at positive bias voltages, that is, when tunnelling into empty sample states, they are clearly visible at negative bias (occupied states). As can be seen in curves 1 through 15 of Fig. 2f (obtained within the box in Fig. 2d), the spectra are characterized by a pronounced peak at −0.2 V, a weaker peak at −0.6 V and a dip at −0.8 V (which are all absent in the relatively featureless spectrum of the Fe monolayer; not shown here). The sequence of spectra reveals that the peak intensity varies periodically. Obviously, the main peak at −0.2 V appears much more intense when the tip positioned above a bright stripe (see, for example, spectrum 2) than above a dark one (spectrum 5).

The sign and intensity of the bias-dependent contrast of the striped superstructure can be analysed more systematically by calculating the energy-dependent asymmetry, which is defined as the difference of the differential conductance measured on and off a bright stripe at a particular energy, *E*−*E*_F_, divided by their sum, that is, (d*I*/d*U*)_on_/(d*I*/d*U*)_off_. The asymmetry curve calculated from tunnelling spectra is plotted in Fig. 2g. Although the asymmetry is negligible at positive sample bias, several features can be recognized at negative bias voltages, with a pronounced asymmetry maximum at −0.2 V.

Figure 2h,i exemplarily show two topographic STM images obtained at the same sample location. Although only subtle modulations can be recognized at *U*=+0.6 V (Fig. 2h), the stripes are clearly resolved at *U*=−0.6 V (Fig. 2i). The corresponding line profiles in Fig. 2j reveal a corrugation of about 14 pm at *U*=−0.6 V, whereas it is below 2 pm at *U*=+0.6 V. Figure 2k summarizes the observed bias dependence of the corrugation. Apparently, the corrugation is extremely low at positive bias, rises up to a maximum value of about 17 pm at *U*≈−0.2...0.4 V—a value that is in line with the above-mentioned energy-dependent asymmetry (*cf*. Fig. 2g)—and then slowly decreases to ≈12 pm at *U*=−1 V.

### Temperature-dependent electronic reconstruction

**Figure 3:**
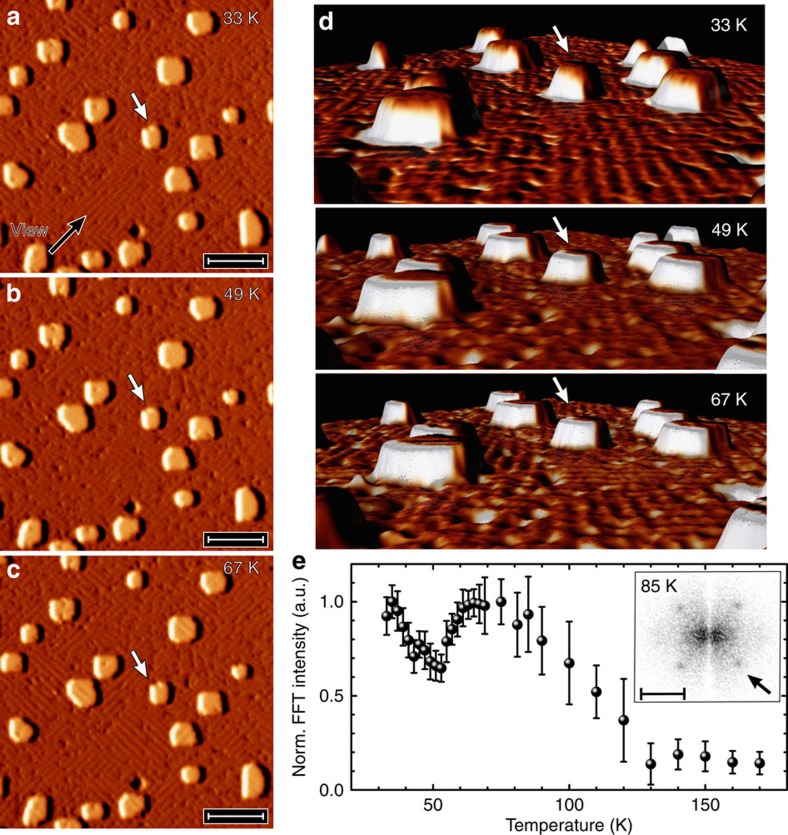
Temperature-dependent charge ordering. (**a**–**c**) STM topographic showing the very same location of the sample surface at *T*=33, 49 and 67 K. Scale bars, 20 nm. One island is marked by white arrows. (**d**) Rendered perspective images as seen along the viewing direction marked by a black arrow in **a**. Although the stripes are clearly visible at low and high temperatures (see, for example, the area in front of the island marked by a white arrow), the intensity is strongly reduced at the intermediate temperature. (**e**) Plot of the temperature-dependent normalized intensity of the spots indicative for the stripe pattern (see arrow). A dip starting at around *T*=70 K can be recognized. An example of a Fourier-transformed STM image taken at *T*=85 K is shown in the inset. Error bars represent the spot's full width at half maximum after subtraction of the background intensity. Scale bar, 1 nm^−1^.

Although the measurements presented so far suggest the existence of two apparently independent ordering phenomena, that is, FM and the formation of stripes with a pronounced local density-of-states modulation, the following data indicate that the two phenomena are coupled and influence each other. Figure 3a–c shows three STM topographic images of a 2-AL Fe film on Rh(001) taken at *T*=33, 49 and 67 K. To exclude any potential influence of local fluctuations of sample quality, all data were taken at the same location as emphasized by white arrows pointing at one particular island. As the rather small corrugation of the stripes indicative for charge order is difficult to recognize, the corresponding rendered perspective images of Fig. 3a–c (viewing direction indicated by a black arrow) are displayed in Fig. 3d. Although stripes are clearly visible at 33 K (Fig. 3a), a much lower corrugation amplitude can be recognized at 49 K (Fig. 3b). Surprisingly, the intensity of the stripes increases again as the temperature is further increased to *T*=67 K (Fig. 3c).

Figure 3e presents a summary of several temperature-dependent data sets that were obtained through fast Fourier transformation (FFT) of constant-current STM images. As can be seen in the inset of Fig. 3e, this results in four spots—one of which is marked by an arrow—indicative of the above-mentioned superstructures with **q**_1,2_. Starting at the lowest temperature accessible with our variable-temperature STM, that is, 35 K, the normalized intensity of these FFT spots first decreases with increasing temperature up to *T*≈50 K. When increasing the temperature further, however, an unexpected upturn of the FFT intensity is observed until approximately the initial value is reached at *T*≈70 K. Raising the temperature beyond this value leads to another reduction of the FFT intensity until it eventually becomes indistinguishable from the background above the charge-order transition temperature *T*_P_=(128±12) K. We note that this temperature dependence is continuous and reversible, that is, the periodic modulations and the spots in the FFT image reappear as the temperature is lowered, consistent with a second-order phase transition and excluding any potential ageing or contamination effects.

Our experimental observations indicate that there are two competing ordering phenomena at work: (i) charge order sets in at ∼130 K and results in stripes visible at negative bias voltages, and (ii) ferromagnetic order, which can be observed by SP-STM below ∼75 K. As both ordering phenomena can be explained by Fermi surface instabilities, some degree of cross-correlation can readily be expected. It should be noted that in many cases the onset of charge order coincides with electronic structure changes, which are not the largest directly at the Fermi level but at slightly different binding energies. For example, when cooling through the charge-density wave phase transition temperatures of 1*T*-TiSe_2_ (refs 30, 31) or 1*H*-TaSe_2_ (ref. 32) in temperature-dependent photoemission spectroscopy experiments, the strongest variation in the energy distribution curves was observed at *E*−*E*_F_=−0.2 eV, that is, at a binding energy similar to what we present in Fig. 2f. We can only speculate why the peak that is indicative of charge order in our STS spectra does not appear directly at, but 200 meV below, the Fermi level. One possibility would be that the responsible bands somewhat disperse and exhibit tunnelling matrix elements that are higher for states, which are still involved in the phase transition, but further away from the Fermi level.

### GL theory

Indeed, the observed phenomenology can be modelled within a GL theory. Its formulation is dictated by the universal properties of the system, such as the number of components of the order parameter and the symmetries of the system. In the present case, there are two order parameters describing the charge and magnetic order. Pinning of the charge order and magnetic anisotropy allow to consider two scalar order parameters: the charge order parameter *ϕ*, which can be identified with the intensity of the FFT spots, and the Ising-like magnetization *m*. The systems exhibits a 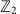 symmetry on both order parameters, *ϕ*→−*ϕ*, *m*→−*m*, so that the global symmetry group is 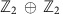. A GL free energy is obtained by expanding the free energy *F* in powers of *ϕ* and *m*, retaining only the terms that respect the given symmetry group (see, for example, ref. 33):


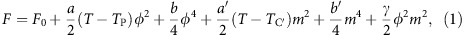


where *γ* is the coupling constant between *ϕ* and *m*, and we have already encoded the expected temperature dependence of the quadratic terms close to the onset of non-zero order parameters.

The minimization of the free energy *F* determines thermal equilibrium, whose stability requires *b*, *b*′>0. Depending on its coefficients, one finds in general four possible solutions to the minimization of *F*: a solution where both order parameters vanish, two solutions where one of the order parameter is vanishing and a solution with a coexistence of both order parameters. The observed charge order in the absence of magnetization implies that *T*_C′_<*T*_P_ and *a*, *a*′>0, so that *ϕ* orders at *T*=*T*_P_. When the coupling constant *γ* satisfies the constraints *ab*′/*a*′<*γ*<*a*′*b*/*a* and *γ*^2^<*bb*′, *ϕ* exhibits a maximum at the magnetic critical temperature *T*_C_=(*a*′*bT*_C′_−*γaT*_P_)/(*a*′*b*−*γa*).

**Figure 4:**
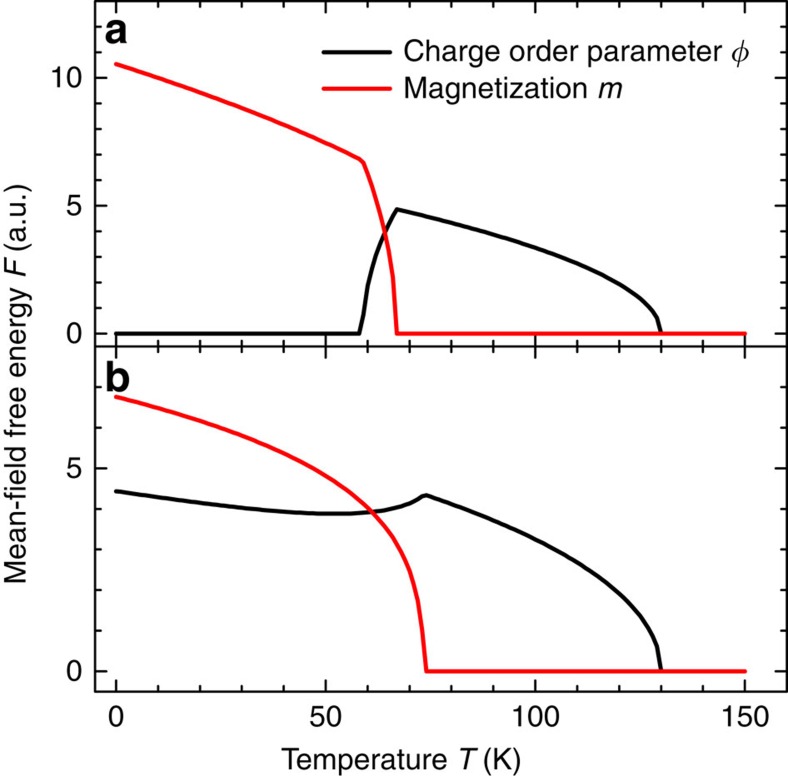
Expansion of the temperature-dependent GL free energy. Plots showing the results of an expansion of the GL free energy *F* to the fourth ((**a**) parameters: *a*=0.9, *a*′=1, *T*_P_=130, *T*_C′_=100, *b*=2.4, *b*′=0.9, *γ*=1.4, *c*=*c*′=0) and to the sixth power ((**b**) same parameters, except for *c*=*c*′=0.015), as described in equations (1) and (2), respectively.

An example of the resulting order parameters is shown in Fig. 4a. We observe that this solution displays an interval of temperature where *ϕ* vanishes, while *m*>0. This behaviour is a result of the competition between the 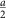 (*T*−*T*_P_)*ϕ*^2^ term, which is negative for *T*<*T*_P_, and the positive coupling with *m*, 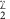
*ϕ*^2^*m*^2^: on decreasing the temperature, *m* grows and a strong-enough coupling *γ* pushes down the value of *ϕ*, which minimizes *F*, eventually leading to *ϕ*=0. However, this zero of the charge-order parameter is unstable with respect to the inclusion of higher-order terms in equation (1). Although such corrections are irrelevant close to the phase transitions of *ϕ* and *m*, they nevertheless influence their growth in a wider range of temperatures. In fact, the expansion of equation (1) up to the fourth order predicts the order parameters to grow indefinitely below the critical temperature, whereas in real materials a saturation effect is expected. This suggests to study an improved GL free energy expansion, including next-to-leading powers:


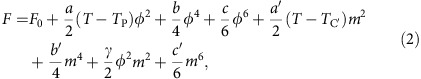


where stability requires *c*, *c*′>0. The inclusion of the sixth power qualitatively changes the behaviour of *ϕ*, giving rise to a dip qualitatively similar to the experimentally observed behaviour. In other words, the solution with a vanishing charge order and non-zero magnetization requires a fine-tuning of the higher-order terms *c*=*c*′=0, whereas their inclusion naturally explains the observed dip in *ϕ*. The sixth term effectively damps the growth of *m* away from *T*=*T*_C_, such that *ϕ* is not pushed down to 0, but instead displays a minimum.

## Discussion

Our experiments show that a sample system that is conceptually as simple as the pseudomorphic Fe double layer on Rh(001) may possess different ordering phenomena. Even more importantly, our data reveal that the ordering phenomena at play here, that is, charge order and FM, compete with each other as evidenced by the intermediate reduction of the charge-order parameter *ϕ* observed experimentally and in GL calculations. We speculate that this cross-talk is caused by the fact that the electronic structure close to the Fermi level, which is responsible for charge- and magnetic-order phenomena through Fermi surface nesting and the exchange interaction, respectively, drastically changes at the respective critical temperatures. We expect that details of the temperature-dependent evolution of the electronic structure will be subject of future experiments such as angular-resolved photoemission spectroscopy, to shed light on the subtle balance between nested and exchange-split electronic states.

## Methods

### Scanning probe microscopy measurements

STM measurements were performed under ultrahigh vacuum (*p*≤5.0 × 10^−11^ mbar) with a home-built low-temperature (LT)-STM and a commercial variable-temperature (VT)-STM at sample temperatures of *T*_LT_=5 K and *T*_VT_=30...300 K, respectively. STM tips were prepared from electro-chemically etched tungsten (W) wires that were flashed under ultrahigh vacuum conditions. For spin-resolved measurements, the W tips were coated with ≈20 ALs of Cr, resulting in out-of-plane magnetic sensitivity, as verified by test measurements on samples with well-known magnetization directions, that is, Fe/W(110) or Mn/W(110)^29^. For STS measurements, a small modulation was added to the sample bias voltage *U* (frequency *ν*=5.777 kHz; amplitude 5 to 15 mV), such that tunnelling differential conductance d*I*/d*U* spectra and d*I*/d*U* maps can be acquired by detecting the first harmonic signal with a lock-in amplifier.

### Sample preparation of Fe/Rh(001)

The Rh(001) surface was prepared by cycles that consist of ∼10 min Ar ion sputtering at room temperature (*p*_Ar_=5 × 10^−6^ mbar, *E*_ion_=1 keV), followed by 150 s annealing at *T*_an_=1,300 K in an oxygen atmosphere and a final flash (duration about 30 s) without oxygen at the same temperature. It has been shown that this procedure reliably removes carbon impurities from the surface^28^. Subsequently, Fe films were grown on the Rh(001) surface at *T*=315 K by means of the e-beam evaporation.

## Additional information

**How to cite this article:** Hsu, P.-J. *et al*. Coexistence of charge and ferromagnetic order in fcc Fe. *Nat. Commun.* 7:10949 doi: 10.1038/ncomms10949 (2016).

## Supplementary Material

Supplementary Information Supplementary Figure 1 and Supplementary References
