# Supplementary material for: Coexistence of charge and ferromagnetic order in fcc Fe
Source: Nat Commun. 2016 Mar 14;7:10949. doi: 10.1038/ncomms10949 (PMC4793077; doi:10.1038/ncomms10949)
Supplement: Supplementary Information — Supplementary Figure 1 and Supplementary References [file ncomms10949-s1.pdf]

SUPPORTING INFORMATION

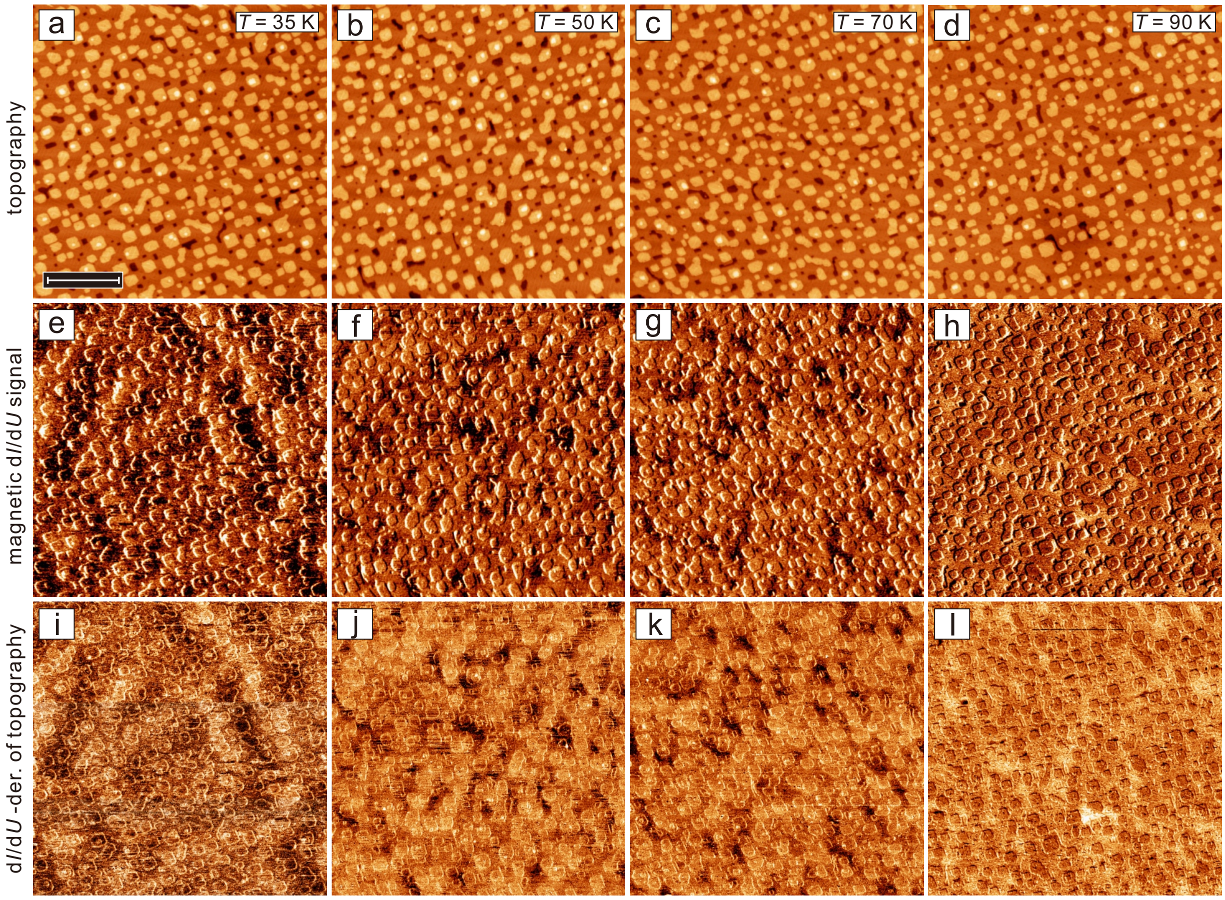

Supplementary Figure 1: **Temperature-dependent magnetic domain structures.** Topography ((**a**)-(**d**)) and the simultaneously measured spin-resolved dI/dU maps ((**e**)-(**h**)) of (2.30 ± 0.05) AL Fe/Rh(001) measured at a sample temperature of $T_S = 35$ K (**a**), 50 K, 70 K, and 90 K (**d**). Scan parameters were $U = -0.7$ V, $I = 500$ pA. Scale bar is 50 nm. An Fe-coated tip sensitive to the in-plane component of the sample magnetization was used. We have tried to emphasize the magnetic contrast by subtracting the derivative of the topography along the fast scan (x)-direction from the dI/dU signal. Thereby, we are able to at least partially compensate the detrimental scan artifact described above. (see (**i**)-(**l**)). While magnetic domains can be recognized at the lowest temperature accessible in our VT-STM, $T_S = 35$ K (**a**), the domain structure is reminiscent to the domain patterns observed previously at low temperature ($T_S = 5$ K) [1]. As the temperature is increased to $T_S = 50$ K and $T_S = 70$ K, the magnetic contrasts become much weaker and the observed domain structure appears more irregular. At the highest temperature of this series,

Supplementary Figure 1: i.e. $T_S = 90$ K, only a very weak magnetic contrast remains, indicating a Curie temperature in the vicinity of 100 K. This result is in excellent agreement with the Curie temperatures T_C determined by Hayashi and co-workers in Fig. 3 of Ref. 2. The spin- and angle-resolved photoelectron spectroscopy data show that T_C steeply decreases as the Fe film thickness is reduced. The slope amounts to approximately 250 K per Fe atomic layer (AL). Extrapolation to 2.3 AL Fe/Rh(001), i.e. the coverage of supplementary figure 1, suggests a $T_C \approx 75$ K, which agrees nicely with the data of Fig. 3 in the main text of our contribution.

SUPPLEMENTARY REFERENCES

[1] Kemmer, J., Wilfert, S., Kügel, J., Mauerer, Hsu, P.-J., & Bode, M. Growth and magnetic domain structure of ultra-thin Fe-films on Rh(001). *Phys. Rev. B* **91**, 184412 (2015).

[2] Hayashi, K., Sawada, M., Yamagami, H., Kimura, A. & Kakizaki, A. Magnetic Dead Layers Induced by Strain at fat Fe/Rh(001) Interface. *J. Phys. Soc. Jpn.* **73**, 2550-2553 (2004).
